# Surveillance of SARS-CoV-2 RNA in open-water sewage canals contaminated with untreated wastewater in resource-constrained regions

**DOI:** 10.1099/acmi.0.000318

**Published:** 2022-01-18

**Authors:** Paramita Basu, Sandeepan Choudhury, Varsha Shridhar, Poorva Huilgol, Samrat Roychoudhury, Indranil Nandi, Angela Chaudhuri, Arindam Mitra

**Affiliations:** ^1^​ Department of Pharmaceutical and Biomedical Sciences, Touro College of Pharmacy, New York, USA; ^2^​ Independent water and wastewater consultant, India; ^3^​ Molecular Solutions Care Health, Bangalore, India; ^4^​ Indian Academy Degree College, Bangalore, India; ^5^​ Jubilant Pharma, Yardley, USA; ^6^​ Swasti Health Catalyst, Bangalore, India; ^7^​ Department of Microbiology, School of Life Science and Biotechnology, Adamas University, Kolkata, India

**Keywords:** open-water sewage canals, untreated wastewater, hyper localized surveillance, SARS-CoV-2, COVID-19

## Abstract

Sewage-based surveillance for COVID-19 has been described in multiple countries and multiple settings. However, nearly all are based on testing sewage treatment plant inflows and outflows using structured sewage networks and treatment systems. Many resource-limited countries worldwide have open canals, lakes and other such waste-contaminated water bodies that act as a means of sewage effluent discharge. These could serve as hyperlocal testing points for detecting COVID-19 incidence using the effluents from nearby communities. However, a sensitive, robust and economical method of SARS-CoV-2 RNA detection from open waste contaminated water bodies in resource-constrained regions is currently lacking. A protocol employed in Bangalore, India, where SARS-CoV-2 RNA levels were evaluated using two open canal systems during the first and second waves in the present study. SARS-CoV-2 RNA was measured using two strategies: a modified TrueNAT^TM^ microchip-based rapid method and traditional real-time reverse transcription-PCR (rRT-PCR), which were compared for analytical sensitivity, cost and relative ease of use. SARS-CoV-2 RNA levels were detected at lower levels during the earlier half compared to the later half of the first wave in 2020. The opposite trend was seen in the second wave in 2021. Interestingly, the change in RNA levels corresponded with the community burden of COVID-19 at both sites. The modified TrueNAT^TM^ method yielded concordant results to traditional rRT-PCR in sensitivity and specificity and cost. It provides a simple, cost-effective method for detecting and estimating SARS-CoV-2 viral RNA from *o*pen-water sewage canals contaminated with human excreta and industrial waste that can be adopted in regions or countries that lack structured sewage systems.

## Introduction

Since the World Health Organization (WHO) declared the COVID-19 outbreak as a pandemic on 11 March 2020 (https://www.who.int), understanding the full extent of the localized incidence and burden of the disease has been an ongoing challenge for public-health officials. There has been a need for systematic surveillance and hyperlocal information or intelligence to direct the response to pandemic control. However, conventional testing, tracing and isolating methods may not be enough to accomplish the desired level of reduction in transmission, morbidity and mortality in very populous countries. This challenge is severe in several developing country settings, such as India, which has the second-highest population globally and has one of the highest population densities in urban areas and lower testing rate capacity per day in a limited well-equipped healthcare facility. Although India has performed the second largest absolute number of rRT-PCR tests for SARS-CoV-2 globally (https://ourworldindata.org), its large population results in one of the lowest tests per 1000 individuals [1–4]. Lack or poor access to water, sanitation and health literacy are major stumbling blocks for implementing basic COVID-19 protocols of frequent handwashing and sanitation, especially in urban areas [5, 6]. Also, contact tracing and exhaustive individual testing in these areas are constrained by a lack of resources and logistics. Thus, there is a need for surrogate indicators for mass testing and identification of emerging infection hotspots, which can assist local public health agencies to make an early response.

Many studies have confirmed the shedding of SARS-CoV-2 RNA from affected symptomatic or asymptomatic patients through the faecal route, enabling wastewater surveillance to capture data on both types of infection [7–10]. The significance of wastewater-based epidemiology was evident with other pathogenic viruses, including polio, Hepatitis A, Hepatitis E, Norovirus and SARS-CoV, which could also be detected in sewage water [11–15]. Untreated wastewater includes waste from household or building use (e.g. toilets, showers, sinks), which contains human faecal waste and waste from non-household sources (e.g. rainwater, industrial use). Untreated wastewater may be sampled from wastewater treatment plant influent (before primary treatment) or upstream in the wastewater collection network. However, in resource-constrained regions, the water in open canals is often contaminated with wastewater and sewage from residential and commercial sources due to a lack of infrastructure or resources or structured sewer systems in several urban and rural areas. Other studies have reported that changes in SARS-CoV-2 RNA concentration levels in wastewater samples collected from wastewater treatment plant influent correlates with reported cases [16, 17]. This data seems to indicate that wastewater surveillance of COVID-19 could be a cost-effective method to survey transmission dynamics of entire communities and collects data from people who may lack access to proper healthcare. It could complement existing epidemiological surveillance methodologies. In addition, depending on the testing frequency, sewage surveillance can be a leading indicator of changes in COVID-19 burden in a community, providing public-health officials with near-real-time information on disease prevalence through infection dynamics earlier than individual diagnostic testing. So, under Indian conditions, COVID-19 can be detected in sewage water. Using the method described here, and when used during routine surveillance, it can serve as an early warning System for the government.

Several research groups in India (in Hyderabad, Pune, Chennai, Bangalore, Gandhinagar) have taken the initiative to join the global efforts for exploring the possibility of using treated sewage and treated wastewater surveillance as a potential tool for early warning for COVID-19 using sewage samples obtained from sewage treatment plants (STPs) [18–24]. However, open urban water bodies containing untreated wastewater that runs very close to dense human settlements are yet to be explored as potential testing sites. In this study, we sought to examine water from open-water bodies such as canals and ponds likely to be contaminated with human excreta and industrial waste from various locations in urban Bangalore to detect SARS-CoV-2. This type of open drainage canal, carrying sewage mixed with stormwater, are common in the developing countries in South East Asia, Africa and Latin America, where structured sewage collection and system is limited. Recently the WHO has emphasized protocols for environmental surveillance for COVID-19 in areas with limited resource availability [25]. Bengaluru, Capital of Karnataka state, the fourth largest city of India with a population of 8.44 million over an area of 709 sq. km has been selected for this purpose (https://bbmp.gov.in/indexenglish.html). Despite having initial control over the number of COVID-19 infected cases since August 2020, Bengaluru had many affected instances [26, 27].

This pilot study reports a simple, cost-effective method for detecting and estimating SARS- CoV-2 viral RNA from open canal sewage sources in regions or countries that lack structured sewage systems. When used routinely, this protocol can detect an early outbreak of COVID-19 infection in areas characterized by high population density and mixed economic characteristics via wastewater surveillance to serve as an early warning system.

## Methods

### Sample collection

We performed this study over the two waves of the COVID-19 pandemic in India in 2020 and 2021. The sample collection was carried in an open canal sewer in Sheshadripuram locality (location 12°59′31.3′′N 77°34′22.2′′E), Gandhinagar ward (Ward no. 94), West Zone, Bangalore Karnataka, India, from an open canal of the Vrishabhavati river, originating from South Bengaluru and flowing South-West cutting across 96 of the 198 wards of BBMP, Municipality of Bengaluru. Sample collection sites were indicated in a Bangalore city ward map in Fig. S1. Once a pristine river and source of drinking water, however, it has now been reduced to an urban drainage canal and designated water quality Category E [28]. This type of water is suitable for irrigation, industrial cooling and controlled waste disposal as per the categorization of the Central Pollution Control Board of India due to the uncontrolled discharge of untreated domestic sewage. It receives almost one-third of the sewage load of the Bengaluru Municipality industrial and agricultural effluent [29]. The Vrishabhavati carries about 500 millions of litres per day (MLD). Only 125 MLD is treated at the Vrishabhavati valley STP.

In 2021, the open canal sewer site sampled was in Doddanekundi (12°58′16.9′′N 77°41′25.9′′E), Ward no. 85, Mahadevapura zone of Bangalore in the eastern part. This canal flows into Doddanekundi Lake. Permission was obtained from BBMP, the municipal corporation of Bangalore urban district, before sample collection. For both the first and second waves, samples were collected on two different days from sites 1 and 2, respectively, during each wave’s earlier and later phases. The first wave of COVID in Bangalore occurred between June and November 2020. Site 1 was sampled during the first wave in its earlier phase (June 2020) and later stage (September 2020). The second wave of COVID in Bangalore occurred between April and June 2021. Site 2 (Doddanekundi) was sampled at two time points during the earlier (May 2021) and later (June 2021) phase of the second wave. A small PET bottle was tied with a string and 200 ml of the sample was collected from the canal. Proper PPE suits were worn during the time of collection. The sample was sealed and transported to the laboratory under cold chain logistics within 1 h of collection, in a styrofoam box with frozen ice packs, for further processing. In the lab, samples were processed within 1 h of receipt. The sample was first inactivated by keeping it under ultraviolet light for 30 min and then pasteurized at 60 °C for 90 min in a water bath. Ultrafiltration of the inactivated sample was done using a 0.2 µm filter into a 50 ml falcon tube containing 4 g of PEG (8000 MW) and 0.9 g of NaCl. The volume was made up to 40 ml. The solution was aliquoted to twenty 2 ml microtubes and centrifuged at 11000 r.p.m. for 30 mins.

### RNA extraction

The resultant supernatant from the step above was discarded, and the pellet was resuspended and pooled into one 2 ml microtube using 600 µl Lysis buffer of QIAamp Viral RNA Mini Kit developed by QIAGEN. The manufacturer’s instruction was followed for the rest of the RNA extraction [30]. The resultant RNA was eluted in 60 µl Elution buffer to a final RNA concentration of 75 ng µl^−1^.

### Detection and estimation of SARS-CoV-2 RNA

The purified RNA was subjected to real-time reverse transcription PCR (rRT-PCR) to detect beta coronaviruses, including severe acute respiratory syndrome coronavirus 2 (SARS-CoV-2). This study used two SARS-CoV-2 detection methods; a newly available MolBio TrueNATlab Duo Real-Time micro-PCR chip-based system and the traditional reverse transcription real-time PCR using Covisure rRT-PCR kit SARS-CoV-2 (Genetix, New Delhi, India) for comparison ease of use and cost-effectiveness.

### TrueNAT chip-based RT-PCR

The initial screening was done using TrueNAT Beta CoV kit, a chip-based rRT-PCR test for the semi-quantitative detection of betacoronavirus *E* gene RNA to estimate beta coronavirus load. The probes used in this kit are designed to target the envelope protein *E* gene sequence from Sarbecovirus and the housekeeping *RNase P* gene from humans. Detection of the constitutive *RNase P* gene serves as an internal positive control (IPC) for all the assays for sample quality, nucleic acid extraction, and PCR. Pre-calibrated *in vitro* transcribed (IVT) RNA for SARS-CoV-2 targeting *E* gene was used as positive quantification control to compare RNA levels detected. Then, 6 µl of RNA solution was pipetted into the chip and inserted in the Truelab Real Time Quantitative micro-PCR Analyzer, for 40 amplification cycles to capture the fluorescent signal displayed as an amplification curve on the analyzer screen, on a real-time basis during the test run. The betacoronavirus ‘DETECTED’ or ‘NOT DETECTED’ result is displayed at the end of the test run. In positive cases, semi-quantitative estimation of the viral load is also displayed on the screen as ‘HIGH’, ‘MEDIUM’, ‘LOW’ or ‘VERY LOW’ based on the cycle threshold (Ct) of the test sample and IVT RNA positive control. Based on the detection of the internal positive process control (IPC), the validity of the test run is also displayed. Following positive detection of betacoronavirus RNA in the initial screening, a chip-based semi-quantitative rRT-PCR confirmatory test was performed by using a TrueNAT SARS-CoV-2 kit to detect SARS-CoV-2 *RdRp* RNA in the extracted RNA sample. The readings were compared with pre-calibrated IVT RNA for *RdRp* target (positive control) to estimate viral load. The sample treatment, detection and analysis procedures are the same as the indicator test described above. The positive controls consisted of a pre-calibrated number of copies genome equivalent of *in vitro* synthesized RNA transcript (IVT RNA) of each target gene (*E* and *RdRp*) included in the rRT-PCR reaction. 4×10^6^ copies ml^−1^ was used for both initial screening and follow-up confirmatory tests.

### Traditional rRT-PCR

We also used the same RNA samples described above for the indicatory test to detect betacoronavirus *E* gene RNA and SARS-CoV-2 *RdRp* RNA by employing reverse transcription real-time PCR using Covisure rRT-PCR kit for SARS-CoV-2 (Genetix, New Delhi, India) as per the manufacturer’s instructions. This kit was used for detection and approximate measurement of *E* gene and *RdRp* of SARS-CoV-2 and human *RNaseP* as internal process positive control. The quantitative positive control (PC) *in vitro* synthesized gene-specific RNA transcripts were also included in the rRT-PCR reaction at 4×10^6^ copies ml^−1^. The PCR ran up to 40 cycles. Primers and probes used in this study are listed in Table 1. Any Ct value above 38 was considered to be negative according to the interpretation of the manufacturer’s guidelines.

**Table 1.** Primers and probe sets used in this study

Assay type	Name	Sequence (5′−3′)	Source
*E* gene indicatory assay	E_Sarbeco_F1	ACAGGTACGTTAATAGTTAATAGCGT	1
	E_Sarbeco_R2	ATATTGCAGCAGTACGCACACA	1
	E_Sarbeco_P1	FAM-ACACTAGCATCCTTACTGCGCTTCG-BHQ	1
*RNase P* gene (internal control) screening assay	RNaseP -F1	AGATTTGGACCTGCGAGCG	1
	RNaseP -R1	GAGCGGCTGTCTCCACAAGT	1
	RNaseP -P1	FAM-TTCTGACCTGAAGGCTCTGCGCG-BHQ	2
*RdRp* gene confirmatory assay	RdRP2_SARSr-F2	GGTAACTGGTATGATTTCG	1
	RdRP2_SARSr-R1	CTGGTCAAGGTTAATATAGG	1
	RdRP2_SARSr-P2 (Specific for SARS-CoV-2)	FAM- TCATACAAACCACGCCAGG-BHQ	2

1 Eurofins Genomics India Pvt. Ltd., Bengaluru

2 Invitrogen, USA

#### Rainfall data

Rainfall data were obtained from the report of Karnataka State Natural Disaster Management Centre (https://www.ksndmc.org/ReportHomePage.aspx). This is based on readings from the rain gauge station located within 1.6 km of the sampling location.

#### Temperature data

Temperature data was obtained from the publicly available records of the Indian Meteorological Society.

#### Community COVID-19 positivity rates data

Community COVID-19 positivity data were obtained from publicly available government gazettes published online in 2020. The number of active COVID-19 patients in the zones under BBMP containing the specific sites of sampling were reported on the four dates the samples were obtained from the data reported in the bulletins of BBMP [31–34].

## Results

### Rainfall and temperature conditions that can affect viral load in open wastewater sample

The rainfall and temperature data in the three consecutive days before the days of sampling are summarized in Table 2. They correspond to the recorded rainfall and temperature readings from the Telemetric Rain gauge station located within 1.6 km of the sampling location available from Karnataka State Natural Disaster Management Centre (https://www.ksndmc.org/ReportHomePage.aspx). On the day of the first sampling set (sample 1), the areas around the sampling location received smaller amounts of rain less than 2.4 mm, but there was moderate rainfall (15–20 mm) in the upstream regions from the sampling location. During the second set of sampling (sample 2), though the upstream catchment received little rainfall during the day of selection, the areas around the sampling location received moderate rainfall in the amounts of 15 mm on the day before sampling. Recorded rainfall data indicates the possibility of dilution of viral RNA load on both days.

**Table 2.** Detection of viral RNA from wastewater TrueNAT RT-PCR and traditional rRT-PCR

Sampling details: date and location	TruNAT RdRp gene detection level indicator results SARS-CoV-2 RNA level	CoviSure RNA rRT-PCR (total cycle no. 40) results SARS-CoV-2 viral load (copy numbers/ml):	Reported burden of COVID-19 in government bulletins (over 3 days prior to the date of sampling)	Average rainfall (over 3 days prior to the date of sampling)	Average temp. (over 3 days prior to the date of sampling)
**17 June 2020 (earlier phase of first wave**) (Location: Sheshadripuram)	very low	10000	144	2.4 mm	26⁰C
**19 September 2020 (later phase of first wave**) (Location: Sheshadripuram)	low	100000	31000	15 mm	27⁰C
**24 May 2021** (**earlier phase of second wave**) (Location: Doddanekundi)	low	70363	40077	1.1 mm	32⁰C
**03 June 2021** (**later phase of second wave**) (Location: Doddanekundi)	very low	5942	128	1.5 mm	31⁰C

June and September have different temperatures and rainfall patterns in Bangalore. June tends to be hotter and less humid, while September is typically the monsoon season with high humidity and lower temperatures. We found SARS-CoV-2 RNA in detectable amounts despite the vagaries of environmental influences on open canal water samples in Bangalore, which is exposed to dilution by rainfall and RNA degradation by atmospheric heat. Interestingly, samples from both sites showed detectable levels of SARS-CoV-2 RNA (Table 2) during both the first and second waves. The RNA was detected even when rainfall diluted the sewage and possible disruption of viral RNA present in the open wastewater due to exposure to a higher temperature (Table 1).

### Detection and semi-quantitative analysis of viral RNA by rRT-PCR

#### Traditional rRT-PCR

**Fig. 1.**
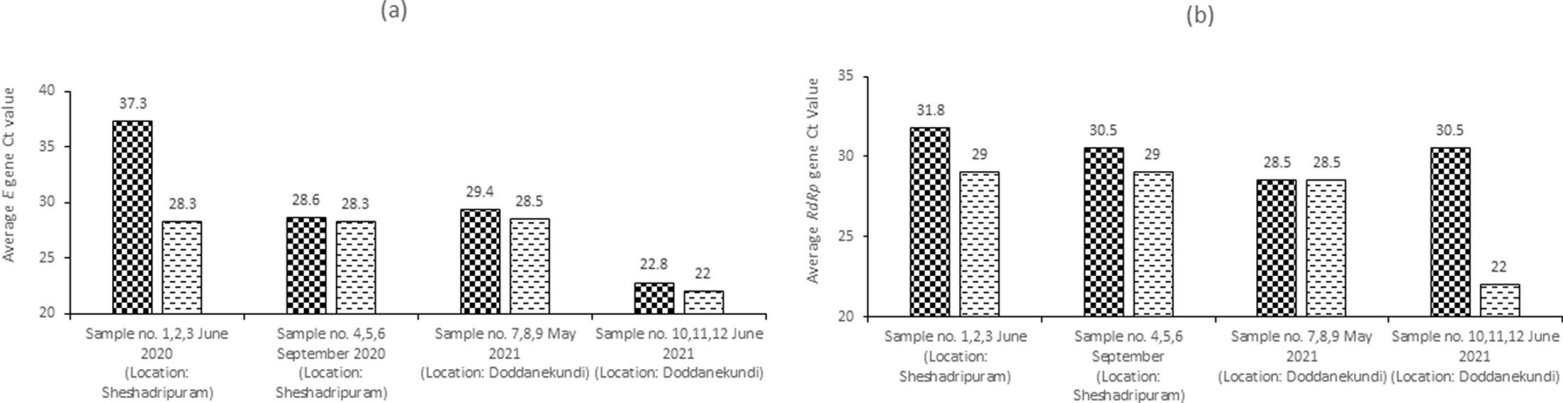
Detection and semi-quantitative analysis of viral RNA by rRT-PCR. (a) Average *E* gene Ct value indicating the presence of betacoronavirus in samples. (b) Average *Rdrp* gene Ct value confirming the presence of SARS-CoV-2 in samples.

Traces of betacoronavirus RNA were observed in both samples 1,2,3 and samples 4,5,6 in the first wave in 2020 as well as samples 7,8,9 and samples 10,11,12 in the second wave in 2021 after the completion of the traditional rRT-PCR. The fluorescence signals obtained from amplification of the target and the internal positive control (IPC) crossed the threshold value in case of both of samples 1,2,3 collected in June 2020, samples 4,5,6 collected in September 2020, samples 7,8,9 collected in May 2021 and samples 10,11, 12 collected in June 2021, indicated detection of betacoronavirus RNA. The PCR amplification was done based on the Envelope gene (*E* gene) of the coronavirus. The difference between Ct values of the IVT RNA positive control (IVT RNA PC) and the average of the Ct values of samples 1,2,3, indicates a 10^3^-fold lower concentration of betacoronavirus RNA in this sample compared to the known positive. However, in samples 4,5,6, samples 7,8,9 and samples 10,11,12, the difference in Ct values between positive control and the average Ct values of the test sample was negligible, indicating that the concentration of betacoronavirus and SARS-CoV-2 RNA in these samples were almost equal to that of the known positive, which is 4×10^5^ copies ml^−1^. No amplification of the negative control was observed. This indicates presence of betacoronavirus in both earlier and latter samples, though level of detected viral RNA is lower in samples 1,2,3 compared to that in sample 4,5,6 (Fig. 1a, Table 2). Since this is an indicator of viral load shed in the collected sample, it seems that viral load seems to have increased in the open canal water from June (earlier phase) to September (later phase) of the first wave.

A confirmatory test was done following the initial screening where amplification was done based on the RNA-dependent RNA polymerase (*RdRp*) gene to positively detect and quantify SARS-CoV-2 RNA. The difference in Ct values between the IVT RNA PC for the *RdRp* gene and the average of samples 1,2,3 reports a tenfold lower SARS-CoV-2 viral RNA concentration compared to the known positive. However, in samples 4,5,6, the difference in Ct values between positive control and the average of the test samples is much less, showing that the concentration of SARS-CoV-2 RNA in this sample is almost equal to that of the known positive, which is 4×10^5^ copies ml^−1^. This confirms the presence of SARS-CoV-2 virus in both earlier (June) and latter (September) phase samples of the first wave in 2020, and the level of detected viral RNA is higher in samples 1,2,3 collected in June 2020 compared to June that in samples 4,5,6 collected in September 2020. This data indicates that the viral load seems to have increased in the open canal water from June to September of 2020. However, in samples 7,8,9 collected in May 2021 at the earlier phase of the second wave, the difference in Ct values between positive control and the average Ct values of the test samples was negligible. Such insignificant differences indicate that the concentration of SARS-CoV-2 RNA in these samples were almost equal to the that of the known positive, which is 4×10^5^ copies ml^−1^. In contrast, in samples 10,11,12, collected in June 2021 at the later phase of the second wave, the difference in Ct values between positive control and the average Ct values of the test samples indicates a 10^3^-fold lower concentration of SARS-CoV-2 RNA in this sample compared to the known positive (Fig. 1b, Table 2).

#### TrueNAT chip-based rRT-PCR

Definitive presence of lower levels of betacoronavirus RNA was indicated in samples 1,2,3 collected in June 2022 and samples 4,5,6 collected in September 2020, and samples 7,8,9 collected in May 2021 and samples 10,11,12 collected in June 2021 after the completion of the PCR cycle. The fluorescence signals obtained from amplification of the target *E* gene RNA and the *E* gene-specific IVT RNA positive control (IVT RNA PC) crossed the threshold value in all samples, which indicated the presence of betacoronavirus RNA (positive detection or ‘detected’). Based on matching the Ct value of the tested samples against the pre-programmed control data into the microchip for automated semi-quantitative viral load estimation, the betacoronavirus *E* gene RNA concentration in the samples 1,2,3 was observed to be ‘very low’. However, in samples 4,5,6, the concentration of betacoronavirus RNA in this sample was observed as ‘detected’ and ‘low’. This indicates the presence of betacoronavirus in both earlier and latter samples, though level of detected viral RNA is lower in samples 1,2,3 compared to that in samples 4,5,6. Similarly, for the samples collected during the earlier and latter phase of the second wave, samples 7,8,9 showed the concentration of betacoronavirus RNA as ‘detected’ and ‘low’ whereas in samples 10,11,12 showed ‘low’. This indicates the presence of betacoronavirus in both earlier and latter samples, though the level of detected viral RNA is higher in samples 7,8,9 from May 2021 than that in samples 10,11,12 from June 2021. The concentration of betacoronavirus RNA was low. Still, it was within the range of positive detection in the initial screening, which indicated the presence of betacoronavirus, the family that includes the novel Coronavirus.

This indicatory data necessitated the follow-up testing to confirm the occurrence of SARS-CoV-2, the causative agent of COVID-19 infection. The presence of lower levels of SARS-CoV-2 RNA was observed in both samples 1,2,3 collected in June 2022 and samples 4,5,6 collected in September 2020 and samples 7,8,9 collected in May 2021 and samples 10,11,12 collected in June 2021 after the completion of the PCR cycle. The fluorescence signals obtained from amplification of the target *RdRp* gene RNA and the IVT RNA quantitative positive control specific for *the RdRp gene crossed the threshold value in all* samples showing positive detection of SARS-CoV-2. In samples 1,2,3, the SARS-CoV-2 RNA concentration was observed to be ‘very low’ while that in samples 4,5,6 the SARS-CoV-2 RNA concentration was observed to be ‘low’. This confirms the presence of SARS-CoV-2 virus in both earlier and later phase samples of the first wave, and the level of detected viral RNA is lower in sample 1,2,3 compared to that in sample 4,5,6. Similarly, for the samples collected during the earlier and latter phase of the second wave, samples 7,8,9 showed the concentration of SARS-CoV-2 RNA as ‘detected’ and ‘low’ whereas in samples 10,11,12 showed ‘very low’.

This pattern of increase in detected betacoronavirus including SARS-CoV-2 RNA levels between samples collected in earlier and later phases of both the first and the second waves is similar to the results obtained from the indicatory screening assay and the rRT-PCR-based testing. Since this is an indicator of SARS-CoV-2 viral load shed in the collected sample, viral load appears to have increased in the open canal water from June to September in 2020 and decreased from May to June in 2021. The levels of SARS-CoV-2 RNA were low but were definitely within the range of positive detection in the confirmatory test, which points to the presence of actively infected people in the surrounding communities inhabiting the catchment of the Vrishabhavati and Doddanekundi drainage canal.

### Comparison between MolBio TrueNAT chip-based micro-RT-PCR system and standard rRT-PCR

In this study, we measured SARS-CoV-2 RNA in open canal samples in two ways, using MolBio TrueNAT and using regular rRT-PCR.

#### Comparison of TrueNAT and rRT-PCR readings

TrueNAT is a battery-operated microchip-based battery-operated real-time PCR machine. It can be used for testing in areas that may have challenges in infrastructure or finding trained technicians. We tested the sewage RNA for SARS-CoV-2 RNA using TrueNAT and the routine rRT-PCR. As shown in Table 2, the two results were concordant, even though the reading from the TrueNAT was not in the form of Ct value but was dependent on the Ct values. Also, the variations in RNA level detected in the open wastewater samples by the TrueNAT system during the different phases of the first and second waves were concurrent with the Ct value-based results provided by traditional rRT-PCR. Both systems show higher levels of SARS-CoV-2 RNA detection when infection burdens in the community were increased and correspondingly lower levels of RNA detection when reported numbers of infection were decreased in the surrounding areas. Therefore, readings obtained from TrueNAT and traditional rRT-PCR systems show similar results and are comparable.

#### Per sample processing cost comparison between MolBio TrueNAT chip based micro-RT-PCR system and standard rRT-PCR

Perceived feasibility and expense of testing have been compared in Table 3. TrueNAT is an automated rRT-PCR system with built-in control data, which eliminates many steps in the rRT-PCR process and lowers the need for highly skilled operators. While a TrueNAT machine has a low throughput and higher per-test costs, the ease of operations and rapidity of results may make it more attractive for routine sampling at specific areas at specific times. As the TrueNAT machine costs significantly less at $11412 than the traditional rRT-PCR machine at $18796, and this method requires less sophisticated laboratory facilities and resources, testing samples eventually cost much less than the traditional rRT-PCR tests. Since the machine is portable, it is beneficial for deploying in interior districts and remote places and collecting and sending swabs for testing in big cities is difficult.

**Table 3.** Perceived feasibility and expense of testing by TrueNAT chip-based micro RT-PCR system and regularly used traditional rRT-PCR

Item	Modified method using TrueNAT	Traditional rRT-PCR-based method
Cost of machine	₹850000 ($11412.22 or €9700.89)	₹1400000 ($18796.6 or €15977.94)
Operating cost	Low	High
Other resources needed:		
BSL of lab	BSL 2	BSL 2
Centrifuge	No	Yes
UPS	No	Yes
Air conditioner	No	Yes
RTPCR tubes/plates	No	Yes
Number of samples that can processed at a time	2	96
Portable	Yes	No
Human resources:		
Min level of training	DMLT	Ideally BSc
Min Time to train	2 h	2–3 weeks
Time to report	2 h	6 h

### Correlation between RNA levels of SARS-CoV-2 in open canal water samples and total COVID-19 positives cases in the communities neighbouring the collection sites

**Fig. 2.**
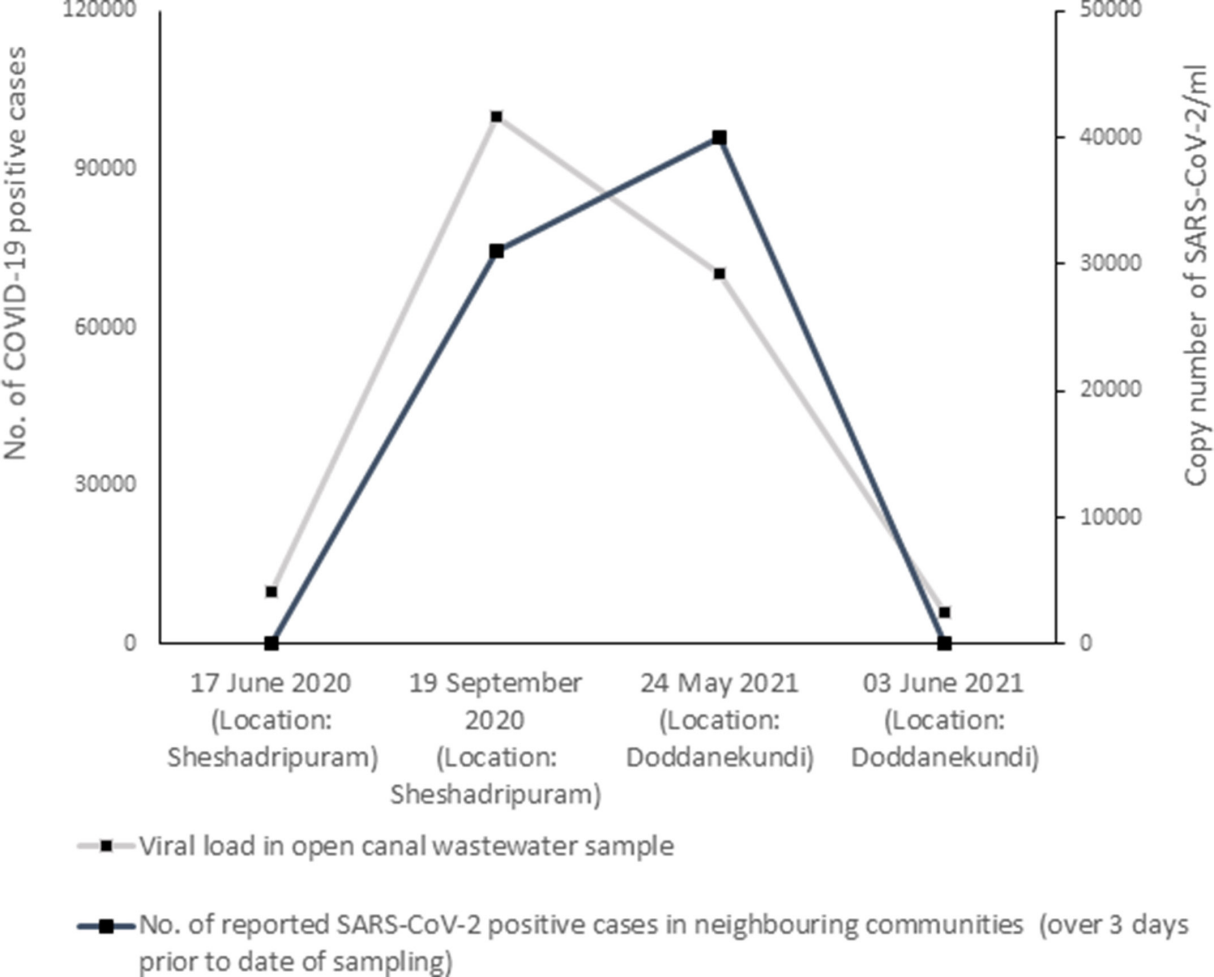
Correlation between COVID-19 community burden and SARS-CoV-2 viral load in open-water sewage canal samples

The reported numbers of confirmed COVID-19 cases in the locality of Sheshadripuram (during the first wave in 2020) and Doddanekundi (during the second wave in 2021), of BBMP Zone West, where both the sample sets were collected on the earlier and later phases [31–34] averaged over 3 days prior to the date of sampling were compared with the copy numbers of SARS-CoV-2 obtained from the samples collected on the corresponding dates to see if there was any correlation between or alignment in the pattern of change of infection burden in the community and viral load in the sewage due to shedding. During the first wave, all the wards in the sewer-shed of the Vrishabhavathi drainage canal near the sampling location had more than 300 active cases when the sampling was done in September, which is much higher compared to that in June 2020. When looking at the correlation between the number of SARS-CoV-2 copies in wastewater and the number of SARS-CoV-2 positive cases in the neighbouring communities at sampling locations, from June to September, the amount of virus in the sewer increases as does the number of positive cases. Thus, the viral loads in the open canal in the ward in June and September correspond to the burden of confirmed positive cases in that ward. In the second wave of 2021, there was a precise alignment between the pattern of changes in viral RNA detected in samples from the earlier to later phase and the changes in the total community positivity burden in the locality of the collection site (Fig. 2). A decrease in the amount of virus was observed in wastewater from May to June, agreeing with the decline in the number of SARS-CoV-2 positive cases reported in the ward (Table 2).

## Discussions

RNA in sewage is known to be affected by temperature and rainfall [35]. Based on our data, viral RNA appears to be stable over the temperatures and time frames involved in travel through the open drains into the canal water since the target Coronavirus RNA was detectable in both samples [31–34]. The indicative detection of beta coronavirus results agrees with the observations from the confirmatory detection of SARS-CoV-2 in the earlier and later phase samples of both the first (2020) and second (2021) COVID-19 waves. The detected SARS-CoV-2 viral load was much lower in the open canal water sample collected in June than in the sample collected from the exact location in September during the first wave. But during the second wave, the viral load was higher in the samples collected in the earlier phase in May than in the sample collected from the same location in June. These changes could be affected by environmental factors because of the open source. This is a significant factor since open surface drains, storm water channels form a part of the informal sewage collection system in developing nations.

The changes in the reported number of confirmed COVID-19 cases in the communities around the locations where both the sample sets were collected during the first wave in 2020 and in the second wave in 2021 both in the earlier and later phases seem to align well with the viral load detected in the corresponding wastewater samples. Recent reports have also stated similar correlations between viral RNA in city wastewater collected from large wastewater treatment plants or sewer networks and the total number of reported or new positive cases observed in the hospitals in the city [36, 37]. The increase in COVID-19 patients in the area of sampling may be reflected by the increase in coronavirus viral RNA levels detected in the canal water. Not all those detected to be COVID-19 positive in the West Zone of BBMP would contribute to the SARS-CoV-2 RNA in the sewage. As ward 94 is predominantly residential, the shedding of viral material is likely from those asymptomatic and undetected or those known to be positive but healthy enough to quarantine at home. Multiple studies have shown that viral loads in wastewater samples may not directly correlate with the number of infected persons in a community or the percent of the population that is infected in that locality [16, 38].

The readings obtained from TrueNAT and traditional rRT-PCR systems were concordant and comparable with each other since both systems showed higher levels of SARS-CoV-2 RNA detection when infection burdens in the community were increased and similarly showed lower levels of RNA detection when reported numbers of infection were decreased in the surrounding localities. This indicates that the TrueNAT^TM^ system may be suitable to use in the absence of resources needed for traditional rRT-PCR without compromising accuracy. In addition, both systems provided results that corroborated well with the trend of changes in the incidence of COVID-19 cases in the local area.

TrueNAT is a point of care assay, which is expected to be of value in the field settings as this method requires minimal laboratory setup, workforce and skill to perform, which is important in low-resource settings [39–41]. In turn, this would reduce the burden on testing sites performing traditional RT-PCR and shorten the turnaround time of reporting of results. The TrueNAT assays requires 45 min, which is much quicker as compared to traditional rRT-PCR, which takes around 4–6 h for the entire process. This shorter run time allows for offsetting the low throughput handling of the TrueNAT system. Since availability of manpower in the form of semi-skilled technicians is abundant in a densely populated country like India, low cost of machinery is an important factor for success of implementation in a densely populated urban area with lower resources.

As TrueNat is a microchip-based method, the standards are built in microchip of the TrueLab Real Time micro-PCR Analyzer. In this system, all the reagents (which works on Taqman chemistry) and a positive control are already preloaded in a cartridge. Test samples are to be dispensed in the reaction well of the chip and inserted into the analyzer. The skill of handling the reagent without cross contamination is completely eliminated. Technicians with less skills and training would be sufficient for this method.

More data on SARS-CoV-2 concentrations in the faeces of infected individuals is needed to understand the relationship between SARS-CoV-2 RNA concentrations in wastewater and how many people in a sewer-shed are infected. Impact of diluting effect of rainfall on the viral load present (possibly due to shedding in faeces) in the open waste-contaminated water body was observed as there were events of consecutive light to moderate rainfalls before and on the days of sampling. During the first set of sampling in June, the storm runoff from the upstream areas of the Vrishabavati open canal drain raises the possibility of dilution of viral RNA leading to the lower levels of RNA detected in sample 1. However, even with a lower number of patients in the upstream area, such rainfall could not negate RNA detection. The rainfall on the day of the second set of sampling in September occurred mainly around the sampling location and was expected to have a less dominant effect of dilution in sample 2, since the upstream catchment received very small amounts of rain. Several studies have reported data on the detection and quantification of SARS-CoV-2 in samples of wastewater taken from sewage treatment plants worldwide [30, 36, 37, 42–51]. Some of these studies have also shown some degree of correlation between viral load present in the sewage collected in an STP and the number of infected patients in the area served by that sewer line [30, 36, 37, 42, 48, 49, 51, 52]. However, this is the first pilot report of sewage testing for COVID-19 using open canals instead of sewer lines or STPs. Testing an open canal near a community that receives sewage from the neighbouring areas could provide a means to receive hyper-local information from that region in a cost-effective manner.

## Conclusions

This pilot study was designed to explore the feasibility of using samples from waste-contaminated open-water bodies to track SARS-CoV-2 virus in a tropical country like India, based on testing of samples collected from an open canal running through a densely populated area in Bengaluru. This method was intended to detect an early outbreak of COVID-19 infection among the habitants at locations lacking a well-structured sewage network, where the waste and excreta from the surrounding residences are conveyed through open sewage drains and discharged into the local water body. This study also indicates the detectability of SARS-CoV-2 viral RNA in samples collected from urban, waste-contaminated open-water bodies despite the possibility of dilution by stormwater. The method is simple and economical and is applicable in the Indian context, characterized by higher population density, heat, humidity, tropical climate, lower resource availability, and variable sanitation facilities. Since a substantial part of the informal settlers rely on community toilets and temporary toilets, such detection gives a comprehensive picture rather than sampling solely from conventional wastewater sources like STPs and inspection chambers. The data reported shows a basic alignment between the quantitative detection of SARS-CoV-2 in open canal water contaminated with waste and the number of cases reported in the surrounding areas. The present method can anticipate outbreaks and evaluate control measures against COVID-19, and roughly estimate the trends of the burden of infected patients, including presymptomatic, asymptomatic, symptomatic, and undiagnosed cases across time. COVID-19 has placed a tremendous economic burden on countries worldwide: in addition to unprecedented healthcare costs, diagnostic costs can be a barrier in rapid testing and tracing of positive cases. Sewage might well prove to be a cost-effective surrogate for community testing. While the current need is for COVID-19 surveillance, sewage surveillance platforms can track other less urgent but equally critical pandemics such as antimicrobial resistance, recreational drug usage, etc.

Accurate predictions of the actual concentrations of viral RNA in the samples cannot be made as the methods used were semi-quantitative. It would not be easy to determine the absolute numbers of positive or infected individuals upstream to the collection point for evaluating the potential correlation between measured viral load and actual viral shedding into the river. Hence, it is not possible to use this method to directly convert concentrations of viral RNA in wastewater to disease prevalence in a community. Besides, multiple variables exist beyond the control in this approach. The spatial and temporal biological variability in viral RNA excretion over time and between individuals, compounded by variability in the sewage-contaminated open drains and untreated wastewater systems across communities, particularly their size, configuration and composition, interpret trends of SARS-CoV-2 RNA levels in sewage a complex task. In addition, trends seen in sewage may or may not correlate with community burdens. However, longitudinal trends of SARS-CoV-2 RNA levels in wastewater can still help complement traditional surveillance methods to understand trends in community transmission.

In conclusion, the study’s purpose was to detect the viral load in open waste-contaminated water sources to improve community-level surveillance and identification of emerging hotspots to facilitate the use of limited public-health resources such as targeted clinical testing. The striking observations made in this pilot study further support the potential use of the described method for monitoring waste-contaminated open urban water bodies, uncovered sewage drains and untreated wastewater for early detection of infections in communities. The absence of easy access to modern diagnostic resources provides an early warning system and guides the initialization of early responses to contain the community spread of COVID-19 in settings with limited resources. While the current need is for COVID-19 surveillance, sewage surveillance platforms can be used to track other less urgent but equally critical pandemics such as antimicrobial resistance or unregulated opioid usage in the community.

## Supplementary Data

Supplementary material 1
