# Supplementary figures and images for: Surveillance of SARS-CoV-2 RNA in open-water sewage canals contaminated with untreated wastewater in resource-constrained regions
Source: Access Microbiol. 2022 Jan 18;4(1):000318. doi: 10.1099/acmi.0.000318 (PMC8895599; doi:10.1099/acmi.0.000318)

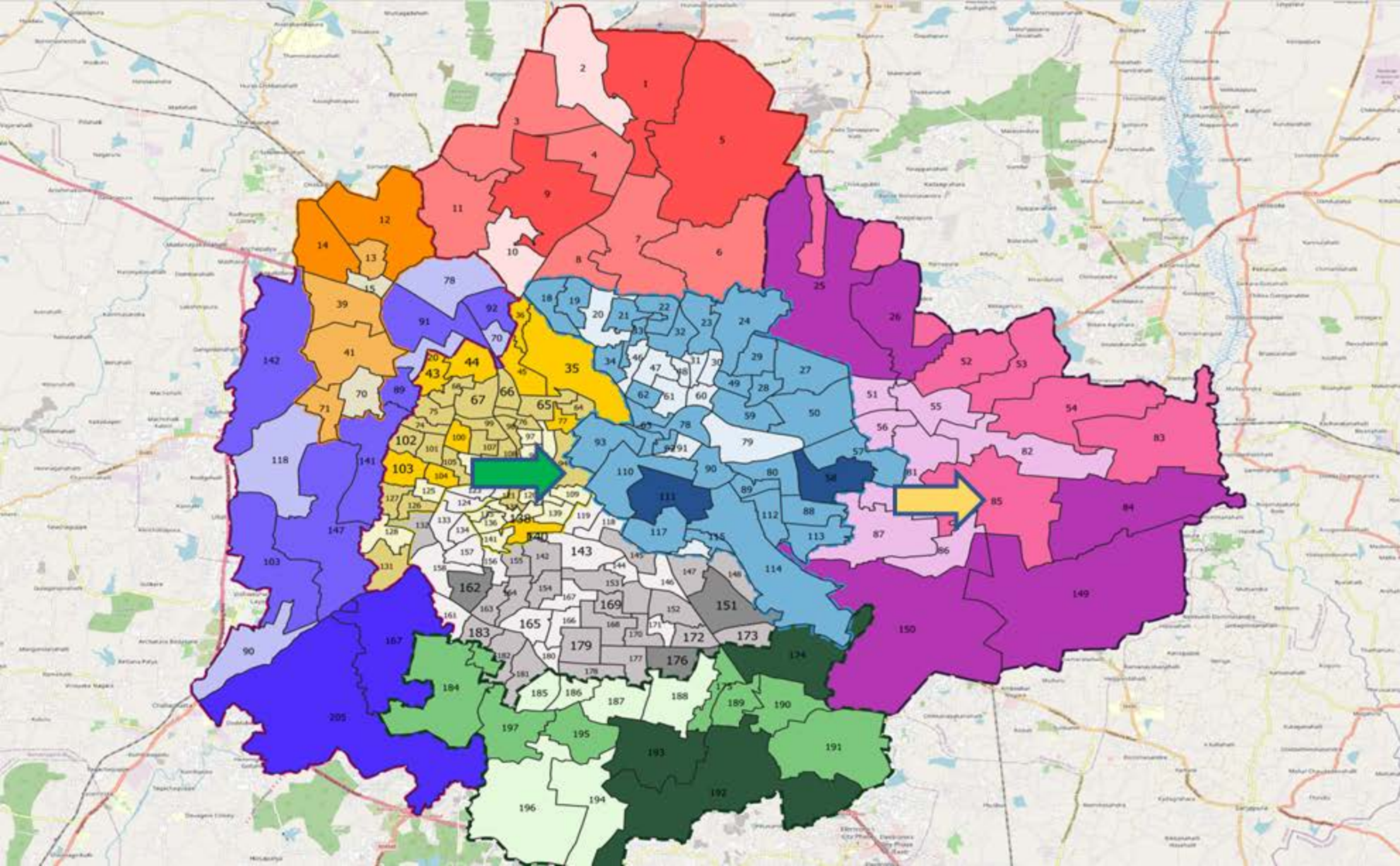

Sampling site  
for first  
wave (2020)

Sampling site  
for second  
wave (2021)

Supplement: Supplementary material 1 [file acmi-4-0318-s001.pdf]
